# The complete chloroplast genome sequence of *Anaphalis margaritacea* var. *yedoensis* (Asteraceae) and phylogenetic relationships within Gnaphalieae

**DOI:** 10.1080/23802359.2022.2048213

**Published:** 2022-04-01

**Authors:** Taishi Hoson, Takuro Ito, Masayuki Maki

**Affiliations:** Botanical Gardens, Tohoku University, Sendai, Japan; Graduate School of Life Sciences, Tohoku University, Sendai, Japan

**Keywords:** Adaptation, complete chloroplast genome, Gnaphalieae, pebbled river banks, phylogeny

## Abstract

*Anaphalis margaritacea* var. *yedoensis* is a perennial herb adapted to the severe environment of pebbled river banks, where it is frequently found. In this study, we determined the complete chloroplast genome of *A. margaritacea* var. *yedoensis* and uncovered its phylogenetic relationships with other members of Gnaphalieae. The total chloroplast genome size of *A. margaritaceae* var. *yedoensis* is 153,231 bp, with a large single-copy region (LSC) of 84,981 bp, a small single-copy region (SSC) of 18,481 bp and a pair of inverted repeat (IR) regions of 24,885 bp. A total of 136 genes were annotated, including 39 tRNA genes, 8 rRNA genes, and 89 protein-coding genes. Phylogenetic analysis showed that *A. margaritacea* var. *yedoensis* and another *Anaphalis* species, *A. sinica*, do not form a monophyletic group, supporting previous phylogenetic studies using some specific regions of cpDNA that showed the genus *Anaphalis* is non-monophyletic.

*Anaphalis margaritacea* (L.) Benth. et Hook. f. 1873 (Gnaphalieae, Asteraceae) is a perennial species with a wide distribution ranging from the Himalayas, across East Asia including Japan, to North America (Meng et al. 2010). *Anaphalis margaritacea* var. *yedoensis* (Franch. et Sav.) Ohwi 1965 is a variety endemic to Japan that has more densely hairy leaves and a better-developed main root than *A. margaritacea* (Kadota et al. 2017). This variety inhabits river banks covered with pebbles exposed to strong sunlight and periodic flooding, and thus the morphological features are thought to be the result of adaptation to the specific environment of its habitat. Genomic information about these tolerance characters may be useful for breeding cultivars belonging to the Asteraceae. Although phylogenetic information about the species is indispensable for such purposes, the taxonomic position of *A. margaritacea* var. *yedoensis* has not yet been elucidated. Thus, our goal for this study was to determine the complete chloroplast genome of *A. margaritacea* var. *yedoensis* and use it to examine the phylogenetic position of the Gnaphalieae.

We collected an individual of *A. margaritacea* var. *yedoensis* from a population (38˚39′06″06 N, 140°52′58″74E) beside the Eai River, Miyagi Prefecture, Japan and cultivated it in the Botanical Gardens, Tohoku University. Collections of the species used in this study are not prohibited by any regulations including our university and regional, national, or international ones. In addition, all the collections were conducted outside legally protected areas. A voucher specimen (Taishi Hoson L8) was deposited in the herbarium of the Botanical Gardens, Tohoku University (TUS; contact Takuro Ito: takuro.ito.c4@tohoku.ac.jp). Fresh leaves were sampled from this cultivated plant and total genomic DNA was extracted using the CTAB method (Doyle and Doyle 1987). The purified DNA was sent to a company (Macrogen: Tokyo, Japan) where it was used for paired-end 150 bp sequencing using the Illumina Hiseq X platform. The raw data (83,137,468 reads) were assembled by NOVOplasty (Dierckxsens et al. 2017) and annotated by Geseq (Tillich et al. 2017). The annotated complete genome sequence of *A. margaritacea* var. *yedoensis* was submitted to DDBJ under the accession number LC656264.

The whole cp genome of *A. margaritacea* var. *yedoensis* was 153,231 bp in length, with a large single-copy region (LSC) of 84,981 bp, a small single-copy region (SSC) of 18,481 bp and a pair of inverted repeat (IR) regions of 24,885 bp. GC content of the whole cp genome was 37.11%. A total of 136 genes were annotated, including 39 tRNA genes, 8 rRNA genes, and 89 protein-coding genes.

To reveal the phylogenetic position of *A. margaritacea* var. *yedoensis* within Gnaphalieae, a phylogenetic analysis was performed with the complete chloroplast genome sequence of *A. margaritacea* var. *yedoensis* and other species of Gnaphalieae including the *Anaphalis* species examined previously (*A. sinica* Hance 1874). Following previous phylogenetic research using three regions of cpDNA (Fu et al. 2016), *Artemisia montana* (Nakai) Pampan 1965 and *Chrysanthemum indicum* (L.) des monl. 1888 belonging to Anthemidiae and Astereae, respectively, were used as outgroups (Figure 1). The sequences were aligned using MAFFT 7 (Katoh and Standley 2013). RAxML version 8.2.12 (Stamatakis 2014) was used to construct the maximum likelihood tree under the TPM1uf + G4 model that was determined to be the best-fitting model by ModelTest-NG (Darriba et al. 2020); bootstrap probability values (BS) were calculated from 1000 replicates.

**Figure 1.**
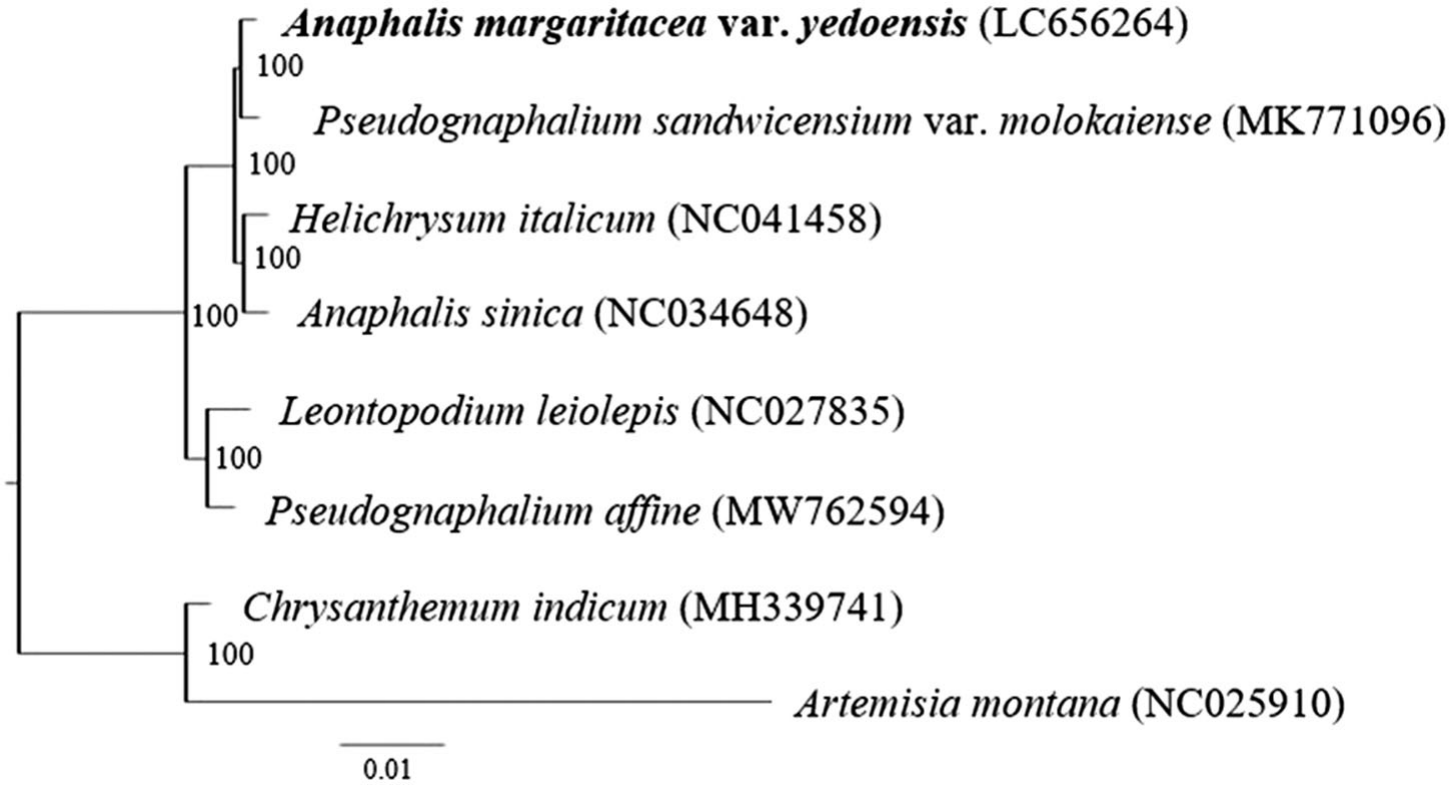
Phylogenetic position of *Anaphalis margaritacea* var. *yedoensis* revealed by a maximum-likelihood tree based on complete chloroplast genomes. *Artemisia montana* and *Chrysanthemum indicum* are included as outgroups. The sample used in this study is shown in boldface type. Numbers at nodes indicate bootstrap support values.

The phylogenetic analysis showed that the two *Anaphalis* species did not form a monophyletic group while it strongly supported the conclusions that *A. margaritacea* var. *yedoensis* and *Pseudognaphalium sandwicensium* var. *molokaiense* (O.Deg. & Sherff) W.L.Wagner, 1999 and *A. sinica* and *Helichrysum italicum* (Roth) G. Don fil. 1830 were monophyletic with very high bootstrap values (both BS: 100). These results support a previous phylogenetic study showing that there are two phylogenetic groups within *Anaphalis* (Nie et al. 2013) and another study showing that the genera *Helichrysum*, *Anaphalis*, and *Pseudognaphalium* are not monophyletic in a cpDNA tree (Galbany-Casals et al. 2014). By contrast, in the latter study, the phylogenetic tree based on nrDNA sequences showed that these genera are monophyletic (Galbany-Casals et al. 2014). We also sequenced ITS and ETS regions of *A. margaritacea* var. *yedoensis* and reconstructed a phylogenetic tree of the taxa closely related to this variety. As the result, the species was included in a clade comprised from other *Anaphalis* species (data not shown), supporting the previous results of nrDNA phylogeny (Galbany-Casals et al. 2014). The incongruence between the phylogenies based on the cpDNA and nrDNA sequences may have resulted from introgression and/or incomplete lineage sorting. In future studies, it will be necessary to test past gene flow among the genera and to reconsider their taxonomic treatments.

## Data Availability

The data that support the findings of this study are openly available in DDBJ (accession no. LC656264) at http://www.ddbj.nig.ac.jp.The associated BioProject, SRA, and Bio-Sample numbers are PRJDB12479, DRA012896, and SAMD00412233, respectively.
